# Parental genetic knowledge and attitudes toward childhood with genetic disorders

**DOI:** 10.3389/fgene.2024.1434322

**Published:** 2024-09-05

**Authors:** Maha Alotaibi

**Affiliations:** King Saud Medical City, Riyadh, Saudi Arabia

**Keywords:** genetic test, parent, genetic counseling, inherited disorders, rare disease, pediatric

## Abstract

**Introduction:** Genetics’ integration with society sparks a multifaceted exploration in medicine, ethics, and psychology. This survey probes parental perspectives on childhood genetic disorders, aiming to gauge their understanding, attitudes, and implications. It seeks to inform healthcare, counseling, and policy endeavors by uncovering gaps in knowledge and attitudes. Understanding the psychological impact and familial dynamics of genetic information underscores the need for tailored support services amidst rapid advancements in genetic technologies and their ethical complexities.

**Methodology:** It is a cross-sectional survey that assesses parental genetic knowledge and attitudes towards childhood genetic disorders. Data is collected by both paper and electronic formats. Data is cleaned in Excel and analyzed in IBM SPSS 29.

**Results:** Our study included 138 participants, predominantly female (71.7%), with mean age 36.01 years (SD = 8.7). Most were Saudi (81.2%), with university education (65.9%). Notably, 73.2% reported consanguineous marriages. Regarding knowledge, 73.2% demonstrated good understanding of genetic disorders of child. Moreover, 47.8% and 34.1% claimed 40%–60% and 34.1% knowledge levels, respectively. Doctors were the primary information source (79.7%). Participants expressed moderate impact of genetic disorders on their child’s life (65.9%) and family dynamics (45.7%). Satisfaction with medical care was high (41.3% rated it as excellent). Challenges accessing healthcare were reported by 52.9%. Positive experiences with genetic disorders were reported by 62.3%, with male participants more likely to report positive experiences (B = 0.888, *p* = 0.041). Improvement areas included treatment availability (39.1%) and advanced medical tests (20.3%). Notably, informing relatives about the genetic disease significantly predicted positive attitudes (B = 1.006, *p* = 0.008). Overall, obtaining information from doctors significantly enhanced knowledge (B = 2.296, *p* = 0.024).

**Conclusion:** Our study shows significant associations between parental knowledge, attitudes towards genetic disorders, and healthcare experiences. It underscores the importance of informed decision-making and targeted interventions to address challenges and improve outcomes in managing childhood genetic disorders.

## Introduction

The relationship between genetics and society has become a more prominent topic of study in the fields of medicine, ethics, psychology, and genetics. As genetic testing and treatments continue to advance, it has become more crucial [1] than ever to comprehend parents’ perspectives regarding hereditary disorders in their children and their degree of genetic awareness. This survey aims to explore the depth of parental genetic knowledge and their perspectives regarding childhood genetic disorders. It seeks to uncover how well parents understand genetic conditions, their potential causes, and implications, as well as their attitudes towards diagnosis, [2] management, and the ethical considerations surrounding genetic testing and intervention.

The advent of new genetic technologies has revolutionized the way we approach the diagnosis and treatment of genetic disorders. These advancements have not only enhanced our ability to identify and manage genetic conditions but have also raised complex ethical and societal questions. Parents, as primary caregivers, play a pivotal role in making informed decisions about genetic testing and interventions for their children. Their understanding and attitudes towards genetic disorders are crucial in navigating the moral dilemmas and decisions that arise in the context of genetic healthcare [3].

The significance of this research lies in its potential to inform healthcare providers, genetic counselors, and policymakers about the current landscape of parental awareness and attitudes. This information is vital for developing targeted educational programs, counseling strategies, and policies that support families navigating the complexities of genetic disorders. Furthermore, by examining the correlation between genetic knowledge and attitudes, this study aims to identify potential gaps in understanding and areas where misconceptions may exist [4].

Moreover, the psychological impact of genetic information on families is profound. Understanding parental attitudes towards genetic disorders is essential for addressing the emotional and psychological support needs of families. The dynamics of genetic information can influence family relationships, decision-making processes, and the overall wellbeing of affected individuals [5]. This survey is structured to gather comprehensive data from a diverse group of parents, encompassing various backgrounds, educational levels, The intertwining of genetics with societal, ethical, and psychological domains underscores the importance of a multidisciplinary approach to understanding and addressing the implications of genetic disorders. As genetic testing and treatments continue to evolve, the necessity to grasp the nuances of parental perspectives on hereditary disorders in their offspring and their level of genetic literacy has escalated [6]. This survey endeavors to dissect the intricacies of parental genetic knowledge and viewpoints concerning childhood genetic disorders, aiming to reveal the extent of parents’ comprehension of genetic conditions, their potential causes and repercussions, as well as their stance towards diagnosis, management, and the ethical quandaries that genetic testing and interventions precipitate [7].

The rapid advancements in genetic technologies have not only propelled forward our capabilities in identifying and managing genetic disorders but have also introduced a plethora of ethical, legal, and social considerations. The decision-making process of parents, who are often at the forefront of opting for genetic testing and interventions for their children, is significantly influenced by their understanding and attitudes towards these genetic conditions. Their choices are pivotal in the ethical discourse surrounding genetic healthcare, making their perspectives essential for ethical deliberations and policy-making in genetic medicine [8].

The essence of this research is to enlighten healthcare providers, genetic counselors, and policy developers about the prevailing state of parental awareness and perceptions. This knowledge is indispensable for crafting bespoke educational initiatives, counseling modalities, and policies that bolster families in maneuvering the intricacies of genetic disorders. By scrutinizing the association between genetic knowledge and attitudes, the study endeavors to pinpoint areas lacking in understanding and where misconceptions might be rife, thus offering a foundation for educational and counseling interventions [9].

Conclusion, the survey considers the psychological and familial impacts of genetic information, aiming to understand how knowledge and attitudes towards genetic disorders influence family dynamics, decision-making, and psychological wellbeing. The manner in which genetic information is processed and acted upon within families highlights the need for psychological support and counseling tailored to the unique challenges posed by genetic conditions [10].

The aim: of this study is to evaluate the level of genetic knowledge among parents and to assess their attitudes toward childhood genetic disorders. This includes understanding how well parents comprehend basic genetic concepts and inheritance patterns, as well as their perspectives on the implications of genetic disorders for affected children and their families. The study seeks to identify gaps in genetic knowledge, explore variations in attitudes based on demographic factors such as education level, cultural background, and personal or family experience with genetic conditions. Furthermore, it aims to assess the impact of parental attitudes on decision-making related to genetic testing, prenatal screening, and interventions for children with genetic disorders. By exploring these aspects, the study intends to inform strategies for improving genetic education for parents, enhancing support services for families dealing with genetic disorders, and guiding policy development to address the needs of this population.

## Methods

This studied employed a cross-sectional surveyed design titled “Parental Genetic Knowledge and attitudes Toward Childhood Genetic Disorders,” Table 1 to assess parental knowledge about genetics and attitudes toward childhood genetic disorders. The surveyed would have distributed to a diverse population of parents with varying backgrounds, included those with and without direct experience with genetic disorders in their children.

**TABLE 1:** Sociodemographic and other parameters of participants.

		Frequency (n = 138)	Percent
Gender	Female	99	71.7
	Male	39	28.3
Age	Mean (SD)	36.01 (8.7)	
	Range	20–61	
Nationality	Non-Saudi	26	18.8
	Saudi	112	81.2
Education	Secondary or less	38	27.5
	University	91	65.9
	Postgraduate	9	6.5
Are you in a consanguineous (related by blood) marriage?	No	37	26.8
	Yes	101	73.2
Have you informed any of your relatives about your child’s genetic disease, or have you kept it confidential?	No	68	49.3
	Yes	70	50.7

### Participants

The target population included parents or guardians of children aged 0–18 years. Participants would recruited from various settings, including genetic clinics, and through social media platforms, to ensured a wide range of socioeconomic, educational, and cultural backgrounds. The studied aimed to enroll a sample size that was statistically significant to allowed for meaningful analysis of the data. We distributed the survey from November 2023 to February 2024.

#### Inclusion criteria

• Parents or legal guardians of children aged 0–18.
• The child had a genetic disease-confirmed diagnosis.
• Ability to read and understand the survey language.

#### Exclusion criteria

• Non-parents or guardians.
• The child did not have a genetic disease-confirmed diagnosis.
• Parents or guardians who are unable to give informed consent.

### Data collection instrument

The surveyed instrument would have designed to measured two main constructs: parental genetic knowledge and attitudes toward childhood genetic disorders. The knowledge component would included questioned assessing understood of basic genetic concepts, inheritance patterns, and common genetic disorders. The attitude component willed explore beliefs and perceptions about the implications of genetic disorders, ethical considerations regarding genetic tested, and supported for affected families. The instrument willed validated through a pilot studied and adjusted based on feedback.

### Data collection procedure

Surveyed would have been distributed in both paper and electronic formats to accommodate different participant preferences and increased response rates. Electronic surveys will be conducted via a secure online platform, while paper surveys will be distributed and collected at participated sites. Informed consent will be obtained from all participants prior to survey administration.

### Data analysis

A comprehensive statistical analysis conducted on the dataset, encompassing both descriptive and inferential methodologies. Firstly, a descriptive analysis conducted to summarize the demographic characteristics of the participants, which included age, gender, and other features. This provided an overview of the studied population. Subsequently, inferential analysed such as Binary Logistic Regression was used to saw the adjusted predictors of Knowledge, Attitude and Experience. Statistical significance is established at a p-valued of 0.05 or lowered and a 95% Confidence Interval. All statistical analysed was executed used IBM’s SPSS Software, version 29.0.0.

### Ethical considerations

The studied will be conducted in accordance with ethical principles outlined in the Declaration of Helsinki. Ethical approval will be obtained from the Institutional Review Board (IRB) prior to studied commencement. Participation will be voluntary, and confidentiality of responses will be maintained.

## Results

Our study “Parental genetic knowledge and attitudes toward childhood with genetic disorders” consists of 138 participants (Table 1). The gender distribution reveals a predominance of females (n = 99, 71.7%) compared to males (n = 39, 28.3%). The mean age of participants is 36.01 years (SD = 8.7), with an age range spanning from 20 to 61 years. Regarding nationality, the majority are Saudi (n = 112, 81.2%), while 18.8% are non-Saudi (n = 26). In terms of educational attainment, most participants have completed university education (n = 91, 65.9%), followed by secondary or lower education (n = 38, 27.5%), and a minority have pursued postgraduate studies (n = 9, 6.5%). A significant proportion of participants (n = 101, 73.2%) report being in consanguineous marriages. Regarding disclosure of their child’s genetic disease, nearly half of the participants (n = 68, 49.3%) have not informed any relatives or chosen to keep it confidential.

**FIGURE 1:**
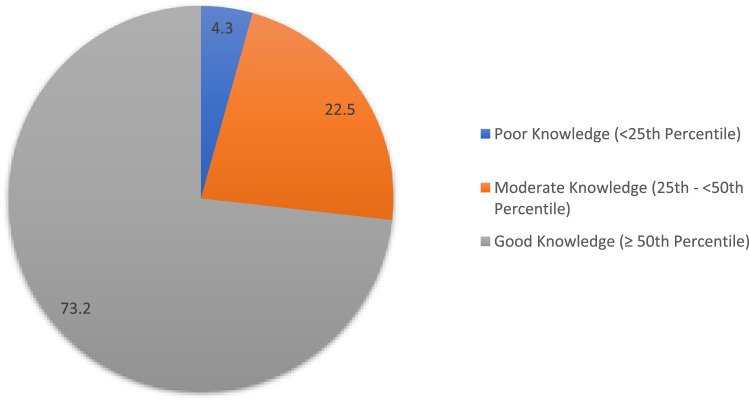
Overall knowledge level about genetic disease of child.

Figure 1 shows the overall knowledge level about genetic diseases among participants. It indicates that a small proportion of participants (4.3%) have poor knowledge, falling below the 25th percentile. A larger segment (22.5%) exhibits moderate knowledge, ranging from the 25th to below the 50th percentile. The majority of participants (73.2%) demonstrate good knowledge, surpassing the 50th percentile.

Table 2 shows the assessment of knowledge and attitudes toward genetic diseases among participants. In terms of knowledge about their child’s genetic disease, the majority report having moderate awareness, with 47.8% (n = 66) indicating a knowledge range of 40%–60%, followed by 34.1% (n = 47) who claim understanding at a level of 80%–100%. Doctors are the primary source of information for most participants (79.7%, n = 110), while a smaller proportion relies on the internet (14.5%, n = 20) or social media (5.1%, n = 7). A significant portion (42.8%, n = 59) feel inadequately informed about their child’s condition, despite 57.2% (n = 79) reporting feeling adequately informed. Additionally, 29.7% (n = 41) know someone with a genetic disease. Concerning attitudes, the majority perceive a moderate impact of the disease on their child’s life (65.9%, n = 91) and family dynamics (45.7%, n = 63). Participants adopt diverse strategies to cope with challenges, including searching for information (30.4%, n = 42), seeking treatment options (39.1%, n = 54), and providing emotional support (30.4%, n = 42). Overall, a positive attitude toward child genetic disorders is predominant, with 61.6% (n = 85) expressing positivity.

**TABLE 2:** Assessment of Knowledge and Attitude towards genetic disease of child.

		Frequency (n = 138)	Percent
Knowledge about genetic diseases of child			
How much do you know about your child’s genetic disease?	10%–30%	25	18.1
	40%–60%	66	47.8
	80%–100%	47	34.1
How did you acquire information about your child’s genetic disease?	Doctors	110	79.7
	Internet	20	14.5
	Social media	7	5.1
Do you feel adequately informed about your child’s genetic condition and its effects?	No	59	42.8
	Yes	79	57.2
Do you know someone with a genetic disease?	No	97	70.3
	Yes	41	29.7
Attitude towards Genetic Diseases of Child			
How do you see the impact of the genetic disease on your child’s life?	No Impact	16	11.6
	Moderate Impact	91	65.9
	Bad Impact	31	22.5
How do you see the impact of the genetic disease on your family dynamics (socially, economically, family relationships)?	No Impact	45	32.6
	Moderate Impact	63	45.7
	Bad Impact	30	21.7
How do you deal with the challenges associated with your child’s genetic disease?	Constantly searching for information and updates about the disease	42	30.4
	Looking for treatment options and specialized centers	54	39.1
	Supporting my child emotionally, psychologically	42	30.4
Attitude Towards Child Genetic Disorders	Negative Attitude (<50th Percentile)	53	38.4
	Positive Attitude (≥50th Percentile)	85	61.6

**FIGURE 2:**
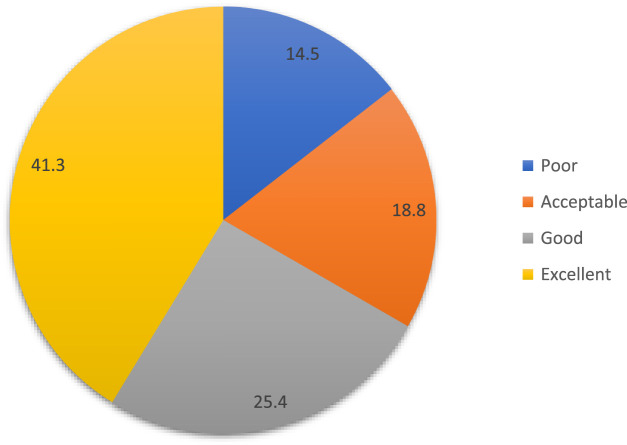
Opinion about the medical care and support available for your child’s genetic disease.

Figure 2 shows opinions regarding the medical care and support available for their child’s genetic disease. A minority (14.5%) perceive the available care as poor, while a slightly larger proportion (18.8%) find it acceptable. A quarter of participants (25.4%) rate the medical care and support as good, indicating satisfaction with the services provided. However, the majority (41.3%) consider the care and support to be excellent, reflecting high levels of satisfaction with the available medical services and support systems for children with genetic diseases.

Table 3 shows participants’ experiences with their child’s genetic disease, perceptions of healthcare services, and future considerations among respondents. The majority express satisfaction with healthcare services (73.2%, n = 101), although a significant proportion has faced challenges accessing them (52.9%, n = 73). Additionally, half of the participants (50.7%, n = 70) report feeling misunderstood or marginalized by healthcare providers due to their child’s condition. However, the overall experience with genetic disorders leans towards positivity, with 62.3% (n = 86) reporting positive experiences. Participants rely on various support systems, including educational support (52.9%, n = 73) and family support (25.4%, n = 35). While many have not explored support groups (51.4%, n = 71), a notable portion have (48.6%, n = 67). Looking ahead, participants express a mix of hopes and fears for their child’s future, with confidence in improvement being predominant (44.9%, n = 62). The overwhelming majority (92.0%, n = 127) express a desire to visit a genetic counselor to discuss preventive measures, highlighting a proactive approach to managing genetic risks in future offspring.

**TABLE 3:** Experience about the genetic disease of child and Perception towards Healthcare system and Future Considerations.

		Frequency (n = 138)	Percent
Experience about genetic diseases of child			
How satisfied are you with the healthcare services provided for your child with a genetic disease?	Unsatisfied	37	26.8
	Satisfied	101	73.2
Have you faced challenges or obstacles while accessing healthcare services for your child?	No	65	47.1
	Yes	73	52.9
Have you ever felt misunderstood or marginalized by healthcare providers because of your child’s genetic disease?	No	68	49.3
	Yes	70	50.7
Experience about Genetic Disorders	Negative Experience (<50th Percentile)	52	37.7
	Positive Experience (≥50th Percentile)	86	62.3
Support System and Resources			
What support systems or resources do you rely on to manage your child’s genetic disease?	Financial Support	30	21.7
	Family Support	35	25.4
	Educational support through Disease awareness	73	52.9
Have you looked into any support groups or online communities for parents of children with genetic diseases?	No	71	51.4
	Yes	67	48.6
Future Considerations			
What are your hopes and fears regarding the future of your child suffering from a genetic illness?	Fearful and anxious	48	34.8
	Confident that my son/daughter will improve for the better	62	44.9
	I cannot determine	28	20.3
Do you want to visit a genetic counselor to discuss appropriate solutions for preventing having children who might inherit the same genetic disease?	No	11	8.0
	Yes	127	92.0

**FIGURE 3:**
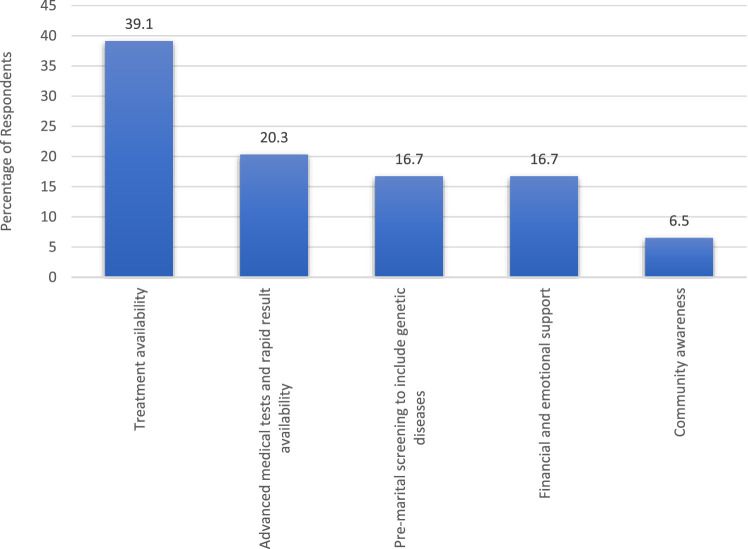
Improvements/Changes needed in the healthcare system to support families affected by genetic diseases.

Figure 3 shows the improvements or changes needed in the healthcare system to better support families affected by genetic diseases. The highest percentage of respondents (39.1%) highlighted the need for improved treatment availability. Following closely, 20.3% expressed the importance of advanced medical tests and rapid result availability. Similarly, 16.7% emphasized the inclusion of genetic diseases in pre-marital screening programs, alongside the necessity for financial and emotional support. A smaller percentage (6.5%) advocated for increased community awareness regarding genetic diseases.

Table 4 shows the adjusted predictors of knowledge and awareness about genetic diseases among participants. The regression analysis indicates several factors influencing knowledge levels. Age shows a negative but non-significant association (B = −0.030, *p* = 0.212, Exp(B) = 0.970), suggesting a marginal decrease in knowledge with increasing age. Gender, nationality, higher education, cousin marriage, and informing relatives about the genetic disease do not significantly predict knowledge levels. However, the source of knowledge is a significant predictor (B = 2.296, *p* = 0.024, Exp(B) = 9.930), with participants obtaining information from doctors being nearly ten times more knowledgeable compared to those relying on the internet and social media.

**TABLE 4:** Adjusted predictors of knowledge and awareness about genetic disease of child.

	B	Sig.	Exp (B)	95% CI	
				Lower	Upper
Age	−.030	.212	.970	.926	1.017
Gender (Male)	.186	.680	1.204	.498	2.910
Nationality (Saudi)	−.759	.196	.468	.148	1.478
Higher Education	−.269	.464	.764	.372	1.570
Cousin Marriage (Yes)	.411	.382	1.508	.601	3.787
Informed Relative about Genetic Disease (Yes)	−.247	.553	.781	.345	1.767
Source of Knowledge (Doctor vs. Internetand social media)	2.296	***.024***	9.930	1.357	72.680
Constant	.425	.784	1.530		

Bold and Italic value represents the significant value <0.05.

Table 5 shows the adjusted predictors of a positive attitude towards genetic diseases in children among participants. Age does not significantly predict attitudes (B = 0.012, *p* = 0.560, Exp(B) = 1.012), suggesting no substantial association between age and attitude. Gender, nationality, higher education, and cousin marriage also do not significantly predict positive attitudes. However, informing relatives about the genetic disease is a significant predictor (B = 1.006, *p* = 0.008, Exp(B) = 2.733), indicating that participants who have informed relatives about the disease are nearly three times more likely to have a positive attitude. The source of knowledge and knowledge about disorders do not significantly predict attitudes.

**TABLE 5:** Adjusted predictors of positive attitude towards genetic disease of child.

	B	Sig.	Exp (B)	95% CI	
				Lower	Upper
Age	.012	.560	1.012	.971	1.056
Gender (Male)	.338	.422	1.403	.614	3.205
Nationality (Saudi)	−.465	.367	.628	.229	1.725
Higher Education	.111	.755	1.117	.557	2.244
Cousin Marriage (Yes)	−.144	.749	.866	.358	2.096
Informed Relative about Genetic Disease (Yes)	1.006	***.008***	2.733	1.300	5.748
Source of Knowledge (Doctor vs. Internetand social media)	.047	.911	1.048	.461	2.384
Knowledge about Disorders	.161	.395	1.174	.811	1.699
Constant	−.982	.432	.375		

Bold and Italic value represents the significant value <0.05.

Table 6 shows the adjusted predictors of a positive experience regarding genetic diseases in children among participants. Age does not significantly predict experiences (B = 0.006, *p* = 0.789, Exp(B) = 1.006), suggesting no substantial association between age and experiences. However, gender is a significant predictor (B = 0.888, *p* = 0.041, Exp(B) = 2.429), indicating that male participants are over two times more likely to report positive experiences compared to females. Nationality, higher education, cousin marriage, informing relatives about the genetic disease, source of knowledge, and knowledge about disorders do not significantly predict positive experiences.

**TABLE 6:** Adjusted predictors of positive experience about genetic disease of child.

	B	Sig.	Exp (B)	95% CI	
				Lower	Upper
Age	.006	.789	1.006	.965	1.048
Gender (Male)	.888	***.041***	2.429	1.038	5.686
Nationality (Saudi)	−.361	.467	.697	.264	1.843
Higher Education	.041	.906	1.041	.532	2.038
Cousin Marriage (Yes)	.292	.501	1.339	.572	3.134
Informed Relative about Genetic Disease (Yes)	.201	.586	1.223	.593	2.523
Source of Knowledge (Doctor vs. Internetand social media)	.090	.829	1.094	.482	2.482
Knowledge about Disorders	.122	.510	1.130	.786	1.625
Constant	−.669	.584	.512		

Bold and Italic value represents the significant value <0.05.

## Discussion

Our study, which has uncovered a complex network of linking of parental knowledge, attitude, and memories concerning genetic disease in their children, shows how these factors relate.

When we take into account the relatively high degree of knowledge among the parents about genetic diseases, this is a good indicator for the tracking of these conditions. The logic behind this is that they would hold the awareness of both the advantages and disadvantages of taking the vaccine which will in turn afford them the privilege of understanding what would be in the best interests of their children (Gallo et al., 2006). Study have proved that the education of the parents about genetic diseases can highly affect the result of the affected newborn since they do the sensitization and adherence to the treatment plans (Woudstra et al., 2022).

The genetic knowledge status of most parents is moderate level, however, suggests that although this information may be widely disseminated, there may be need for further information delivery improvement. The wearing off the healthcare party profession at the source of information that manifests is not alienating to literature declaring critical importance of doctors in patient education. While it is essential to take into account the opinions of parents who advise their children on healthy eating, it is also necessary to develop more detailed communication strategies for which the majority of them are inadequately informed (Houwink et al., 2011; Pelentsov et al., 2015).

In other words, despite the obstacles to being born with genetic disorders, which is the case for several of the participants, their positive outlook is a sign of resilience. Apart from this, being optimistic about the situation could play a role which is proved by multiple studies to improve both family’s psychological health and adherence to treatment regimens. The studies have points out the significance of psychosocial support in dealing with genetic disorders by highlighting that developing positive attitudes also crucially contributes to the medical care (Firth et al., 2022; Kulkarni et al., 2023).

Providing high-quality medical care and other support systems that have been and continue to be crucial indicators of service quality effectiveness. On the other hand, a minority of people who see in the treatment whole inadequacies ascribe the causes of the disparities in the quality of care given. The possible reasons that have been indicated for such an unfortunate incidence include but not limited to geographical location, socioeconomic status and accessibility of specialized services. The barrier for these patients is the limited access to high-quality care (Evans et al., 2022).

Patients’ wish to learn more about their genetic risks and management, including preemptive health measures, is an evident trend. This is what the experts are now emphasizing on the global health structural shift in the move to pick up the early intervention and to assess risks so that one can get better health outcomes (Wojcik et al., 2020). Patients attending genetic counseling could not be considered passive recipients of the account of their genetic tests, their steps could be seen as a sign of their desire to understand and manage the genetic risks, which is indeed of significant importance for the turn that awaits the genetic diseases’ prevention activities.

Referring to knowledge levels being based on the source of the information; one can say that the accuracy and reliability of the counselling are crucial factors in the therapist effectiveness. In line with this evidence, the healthcare professionals are the most important link in patient education process and their role in passing information is essential in order to arm the patient with knowledge and empowerment (Wolyniak et al., 2015). By the inclusion of relatives in genetic disease not only as predictors of positive attitudes but also as social supports for the management of health issues understanding genetics have become increasingly important. Literature generally suggests that a concern person who possess information about can even provide such relevant practical help to the patient and that may change the attitude and coping strategies (Niyibizi et al., 2023).

The gender notice in positive experiences may represent the cultural or social impacts that influence how the genetic conditions are apprehended and cared. It seems that the measure implies a gender specific planning should be in place in order to ensure gender neutral support and care through all parental caretaking. Accessibility and being misread by the healthcare provider are two key issues that have been consistently stated to be barriers in healthcare utilization (Clemente et al., 2022). Such issues can cause a stressful life for these families and they may also feel as if they can’t relate with the health system. Genetic disorders then call for health awareness programs that are tailored to guarantee inclusivity and sensitivity in addressing the specific needs of families battling genetic diseases.

Not being able to manage the disease on their own and the necessity of educational and family support system points out that the care, which the families with genetic disease deal with, have many dimensions to it. It is the hallmark of the general conceptualization of health which is holistic in its approach and it involves the need to address not just the medical concerns but also the emotional and educational demands of patients and their family members (Zamanzadeh et al., 2015). The finding that a number of participants never tried engaging with support groups leaves room for speculation reasoning that such resources may not be as available or accessible as they should be. According to certain studies, support groups have been characterized as the providers of valuable peer support and informational sharing, both of which are necessary for the families dealing with the complexities of genetic problems (Plumridge et al., 2012).

Parents of the baby to face the blurry vision with the mixture of hopes and anxieties for the baby’s future which is due to genetic disease uncertainty. Hope prevails over the sway of strong belief in recovery, which evokes a positive attitude that can be the manager of searching for and implementation of treatment. Increasing number of people seeking advice on genetics reveals a proactive stance to managing genetics-related risks, which is one of the most crucial components of good health in generations to come (Wöhlke and Perry, 2021).

### Clinical implications

We are aware that a large part of the population relies on health professionals for information which therefore becomes a reminder of the great role those professions undertake in finding the truth and support persons. Aiming more family education and engagement to topics covered in this study leaves a room for more in-depth research. It seems that the level of information of the relatives is directly proportionate to the successful attitudes towards genetic disorder management. Healthcare suppliers should make planned information sharing strategies as well as provide an open environment for family discussions so as to promote an informed decision-making positively about managing genetic conditions.

### Future research

The follow up domain of the study could be related to identifying information needs and choices of parents and caregivers of children with this type of genetic disorders. Addressing the problem of insufficient parenting knowledge by evaluating the effectiveness of various educational formats like counseling or web-based resources can be successful in developing programs that help parents get what they need. Surveying the whole-life psychology of families who live with genetically inherited diseases highlighting the diverse set of issues that families dealing with them live with, including coping strategies or resilience can reveal a lot about how to best care for them holistically. Longitudinal studies that periodic family experiences and requirements can generate essential data help to define the best ways for healthcare systems and support systems to services to be provided continually for this population.

### Limitations

Despite the numerous strengths of our recent study, there is some possible limitations that requires acknowledgment. The extent to which the results could be generalized may be determined by the sample size and demographics of the sample, as the study is entirely focused on a specific population or a particular geographic region. The usage of self-report measures in assessing knowledge, attitudes, and experiences presents the possibility of response bias and social desirability bias, which can lead to a situation where results can be swayed by these elements. The cross-sectional nature of the study obviates the determination of the variables’ causal association and renders the analysis of the developing trends and changes over time inefficient.

## Conclusion

Parents know genetic diseases comprehensively, mixed with different conditions and emotions through an investigation. Despite the fact that most people have a justified standard of understanding of health issues, there are still some forms of healthcare that are not readily accessible, and positive experiences vary. The gender based of reporting the positive experience underline those areas that might be given special consideration. It is imperative to highlight that engendering relative about the topic of genetic diseases noticeably resonates with such positive attitude. This study illustrated the necessity of aimed programs to handle access issues and help build support systems in healthcare. The researchers revealed significant information that healthcare providers, policymakers, and genetic counselors can exploit to provide better services, equip people with knowledge to make informed decision-making and guide affected families to navigate through the chaos.

## Data Availability

The datasets presented in this study can be found in online repositories. The names of the repository/repositories and accession number(s) can be found in the article/Supplementary Material.
